# EDAG promotes the expansion and survival of human CD34^+^ cells

**DOI:** 10.1371/journal.pone.0190794

**Published:** 2018-01-11

**Authors:** Ke Zhao, Wei-Wei Zheng, Xiao-Ming Dong, Rong-Hua Yin, Rui Gao, Xiu Li, Jin-Fang Liu, Yi-Qun Zhan, Miao Yu, Hui Chen, Chang-Hui Ge, Hong-Mei Ning, Xiao-Ming Yang, Chang-Yan Li

**Affiliations:** 1 State Key Laboratory of Proteomics, Beijing Proteome Research Center, Beijing Institute of Radiation Medicine, Beijing, China; 2 Tianjin University, School of Chemical Engineering and Technology, Department of Pharmaceutical Engineering, Tianjin, China; 3 An Hui Medical University, Hefei, China; 4 Guang Dong Pharmaceutical University, School of Pharmacy, Guangzhou, China; 5 Department of Hematopoietic Stem Cell Transplantation, Affiliated Hospital to Academy of Military Medical Sciences, Beijing, China; EFS, FRANCE

## Abstract

EDAG is multifunctional transcriptional regulator primarily expressed in the lin^lo^c^-^kit^+^Sca-1^+^ hematopoietic stem cells (HSC) and CD34^+^ progenitor cells. Previous studies indicate that EDAG is required for maintaining hematopoietic lineage commitment balance. Here using *ex vivo* culture and HSC transplantation models, we report that EDAG enhances the proliferative potential of human cord blood CD34^+^ cells, increases survival, prevents cell apoptosis and promotes their repopulating capacity. Moreover, EDAG overexpression induces rapid entry of CD34^+^ cells into the cell cycle. Gene expression profile analysis indicate that EDAG knockdown leads to down-regulation of various positive cell cycle regulators including cyclin A, B, D, and E. Together these data provides novel insights into EDAG in regulation of expansion and survival of human hematopoietic stem/progenitor cells.

## Introduction

Hematopoietic stem cells (HSCs) can give rise to all types of mature cells within the blood and immune systems. Umbilical cord blood (UCB) is an alternative HSC source for allogeneic hematopoietic cell transplantation[1]. However, low absolute numbers of hematopoietic stem and progenitor cells (HSPCs) within a single cord blood unit has remained a limiting factor for this transplantation modality, particularly in adult recipients[2, 3]. Many research efforts have been devoted to exploring UCB expansion strategies.

Erythroid differentiation-associated gene (EDAG) which is homologous to mouse Hemgn[4] and rat RP59[5, 6], is a hematopoietic-specific transcriptional regulator involved in cell proliferation, differentiation and apoptosis[7–9]. In mice, Hemgn is primarily expressed in the lin^lo^c^-^kit^+^Sca-1^+^ HSC population and CD34^+^ progenitor cells in adult bone marrow and down-regulated in mature blood cells[4]. Overexpression of EDAG in mice led to enhanced myeloid development and suppressed lymphoid lineage development[9]. In human UCB CD34^+^ cells, overexpression of EDAG induces erythroid differentiation of CD34^+^ cells in the presence of erythropoietin (EPO) through recruiting p300 to modify GATA1 acetylation[10]. Furthermore, in murine Hemgn is a direct target of HOXB4 and promotes bone marrow cells expansion and self-renewal[11]. However, the role of EDAG in the expansion and survival of human HSPCs remains unknown.

In this study, we examined the role of EDAG in human cord blood (CB)-derived HPSCs. Our data demonstrated that EDAG overexpression enhances the proliferative potential of human CB CD34^+^ cells, increases survival, and promotes their repopulating capacity. Moreover, EDAG overexpression induces rapid entry of CD34^+^ cells into the cell cycle and prevents cell apoptosis. Knockdown of EDAG leads to down-regulation of various positive cell cycle regulators. Taken together, these data indicate that EDAG is crucial for human HSPC expansion and survival.

## Materials and methods

### Isolation and expansion of CD34^+^ cells

Human umbilical cord blood (UCB) units were collected from normal, microbiologically screened and ethics-cleared donors with informed consent of the mothers. All investigations were approved by local Human Research Committees. The participants have provided their written informed consent. Human CD34+ cells were enriched from UCB by magnetic bead positive selection using Miltenyi immunomagnetically activated cell sorter (MACS; Miltenyi Biotech,Auburn, CA). The CD34^+^ cells were then stained for CD45 and the CD34^+^ purity was more than 95% reanalyzed by FACS.

Expansion of the CD34^+^ cells was performed in serum-free medium (SFEM) (Stem Cell Technologies, Cat#09650) supplemented with 100ng/ml rhSCF, 50ng/ml rhIL-3, 50ng/ml rhFlt3-Ligand, and 50ng/ml rhTPO which were purchased from Peprotech.

### Lentiviral virus production and infection

EDAG lentivirus and shRNA lentivirus particles production were performed as previously described[10]. A full-length EDAG cDNA was cloned into lentivirus vector FUGW which generates a EDAG-GFP fusion protein. Full-length EDAG was also cloned into the pBPLV vector, which has two CMV promoters and an IRES-GFP tag. The recombinant vector pBPLV-EDAG expresses EDAG protein and GFP protein simultaneously. For construction of lentivirus-mediated RNA interference, the siRNA sequences were cloned into a psicoR-IRES-GFP vector to generate siEDAG lentivirus. The siEDAG lentivirus expresses CMV promoter-driven GFP protein and U6 promoter-driven siRNA targeting EDAG. For infection, CB CD34^+^ cells were prestimulated in SFEM medium containing 100 ng/ml rhSCF, 50 ng/ml rhFlt3-Ligand, 50 ng/ml rhTPO and 50 ng/ml rhIL-3 for 24 hours and then plated in Retronectin-precoated plate (TAKARA, Cat#T100B). Cells were transduced with lentivirus at the MOI of 10 in the medium containing the same cytokines and 8μg/mL polybrene and centrifuged at 600g for 1 hours under room temperature. After 3 rounds of transfection within 24 hours, cells were collected for FACS sorting or succedent procedure.

### Antibody staining for FACS

Cells resuspended in PBS were stained for different FACS antibodies and subsequently incubated in dark under room temperature for 20 minutes. Then cells were washed and analyzed by FACS Fortessa. CD34-APC(Cat#17–0349)/PE-Cy7(Cat#25–0349), CD71-APC(Cat#17–0719)/ PE-Cy7(Cat#25–0719), Glycophorin A(GPA)-APC(Cat#17–9987), B220-APC(Cat#17–0452), CD19-PE(Cat#12–0199), CD3-PE-Cy7(Cat#25–0038), CD41-PE-Cy7(Cat#25–0419), CD14-PerCP-eFluor610(Cat#61–0149)/PE-Cy7 (Cat#25–0149), Annexin V-APC(Cat#88–8007), and human CD45-PE (Cat#12–9459) antibodies were purchased from eBioscience; Glycophorin A (GPA)-PE(Cat#555570), CD11b-BV421(Cat#562632)/BV605(Cat#562721), CD19-BV605(Cat#562653) were purchased from BD Biosciences.

### In vitro hematopoietic colony forming cells(CFC) assays

CD34^+^ cells transduced with indicated lentivirus were seeded in semi-solid methylcellulose medium (MethoCult® GF^+^H4435, Stem Cell Technologies) according to manufacturer’s recommendation. After 2 weeks of culture the colony number was counted. For secondary replating, the first CFC colonies incubated for 2 weeks were transfered in 250 μL Iscove Modified Dulbecco medium (IMDM) to form single-cell suspensions, and then replated into methylcellulose medium (MethoCult® GF^+^ H4435). The secondary colonies were counted after another two weeks. Colony growth results were indicated as mean (of triplicate plates) ± S.D. colonies per 1000 cells plated.

### Long-term culture initiating cell (LTC-IC) assay

A modified LTC-IC assay was performed. Briefly, 3×10^4^ CD34^+^ cells transduced with indicated lentivirus were cultured MS5 stromal cells in T25 flasks for 4 week in StemSpanTM SFEM serum-free medium (Stem Cell Technologies, Cat#09650) with 100ng/ml rhSCF, 50ng/ml rhTPO, 50ng/ml rhIL-3, and 50ng/ml rhFlt3-Ligand. At weekly intervals, 50% of culture media were replaced. After 4 weeks of culture, the cells were harvested and transferred to methylcellulose medium (MethoCult® GF^+^H4435, Stem Cell Technologies) for colony-forming assays. Colonies were typically scored after 10~12 d of culture.

### NOD/SCIDIL2Rγ^null^ mice reconstitution assay

Freshly separated CD34^+^ cells were cultured for 24 hours in serum-free expansion medium and then infected with lentivirus for 3 times within 24 hours. At the end of the last infection, 2.5×10^5^ of transduced cells were injected intravenously into female NOD/SCIDIL2Rγ^null^ recipient mice of 4~6-week-old which received sublethal irradiation (X-Ray with a total dose of 2.5Gy). At 16 weeks after xenotransplantation, six recipients were sacrificed by cervical dislocation. Animal experiments were performed with approval of the Institutional Animal Care and Use Committee of Beijing Institute of Radiation Medicine (IACUC-2014038).

### Protein extraction and Western blotting

Cells were lysed with M-PER Mammalian Protein Extraction Reagent (Pierce, Rockford, IL,USA). Protein concentrations were determined using BCA protein assay (Beyotime). Total protein (10ug) was separated by SDS–PAGE and transferred to polyvinylidene difluoride membranes (Millipore). Membranes were probed with specific primary antibodies, antibody–protein complex detected by horseradish peroxidase-conjugated secondary antibodies and enhanced chemiluminescence (ECL) exposed (Pierce). Anti-EDAG(H-300) sc-68361 and anti-GAPDH(N-15) sc-584 antibodies were purchased from Santa Cruz Biotechnology.

### Real-time PCR analysis

Total RNA was pre-treated with Dnase and reverse-transcribed, and amplified using the Transcriptor High Fidelity cDNA Synthesis Kit (Roche). Real-time RT-PCR reaction was performed by ABI7500 (Aapplied Biosystems, U.S.). The abundance of mRNA of each gene was normalized to GAPDH. The qPCR primers are listed in S1 Table.

### Statistical analysis

Error bars represent the mean ± SD determined from three separate experiments and the statistical significance between the means was determined using a two-tailed t-test.

## Results

### Overexpression of EDAG promotes the proliferation and maintenance of human CB CD34^+^ cells

**Fig 1:**
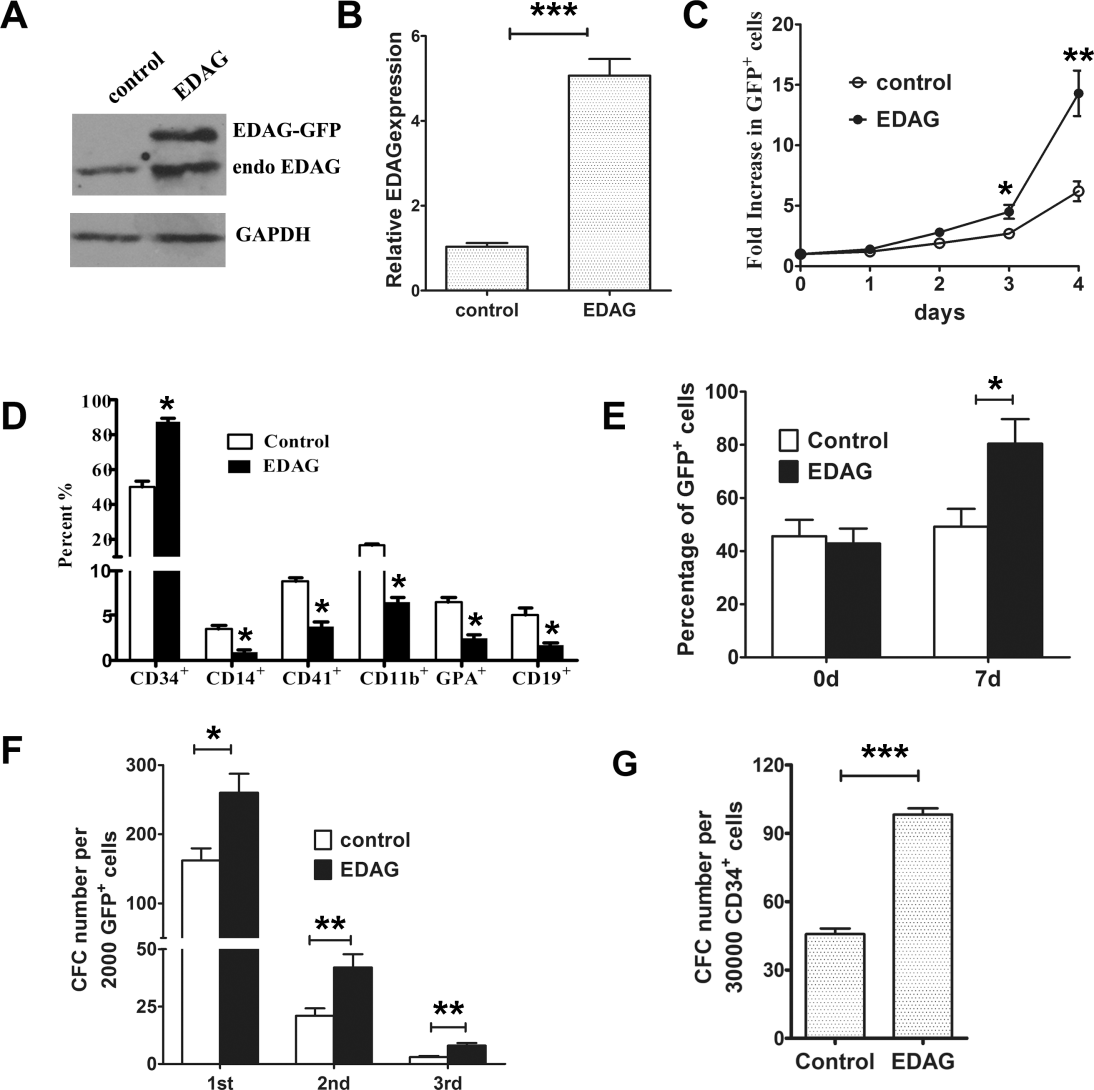
Overexpression of EDAG promotes the proliferation and maintenance of human CB CD34^+^ cells. (A) Validation of EDAG overexpression in CD34^+^ cells infected with EDAG lentivirus. EDAG lentivirus was infected into CD34^+^ cells and the expression of EDAG was analyzed by Western blot with EDAG antibody. GAPDH was used as internal control. Total RNA was extracted for Real-time PCR analysis (B). (C) EDAG or control lentivirus were transduced into CB CD34^+^ cells and GFP-positive cells were sorted. Then cells were plated in liquid cultures for the indicated time and the number of cells was counted. At 7 days, the expression of the indicated surface markers were analyzed by FACS (D). (E) EDAG or control lentivirus were transduced into CB CD34^+^ cells and nonsorted cells were cultured for 7 days. The percentage of GFP^+^ cells was analyzed by FACS. (F) CFC activity of CB CD34^+^ cells transduced with EDAG or control lentivirus with serial replating. (G) LTC-IC assay in bulk T25 flasks. Data are shown as the mean ± S.D and are representative of three independent experiments. * *p*<0.05, ** *p* < 0.01, *** *p* < 0.001.

We first investigated the effect of EDAG overexpression on the proliferation of human CB CD34^+^ cells. EDAG or control lentivirus were transduced into CB CD34^+^ cells and GFP-positive cells were sorted. The expression of EDAG protein was confirmed by Western blotting analysis. About 1.2-fold increased exogenous EDAG (EDAG-GFP) expression level was observed compared to the endogenous EDAG (Fig 1A, S1 Fig). This result was confirmed by Realtime RT-PCR analysis (Fig 1B). Then the sorted cells were plated in cytokine-driven stroma-free cultures for daily analysis. As shown in Fig 1C, control cells expanded 6~fold within 4 days, and EDAG overexpression resulted in a strong expansion of more than 14~fold, suggesting that EDAG promotes the proliferation of CD34^+^ cells. FACS analysis revealed that about 90% of the EDAG overexpressing cells remained CD34 over a period of 7 days, whereas in control cells only 50% cells were remained (Fig 1D). The differentiation markers for myeloid (CD11b, CD14), lymphoid (CD19), Megakaryocytic (CD41) and erythroid lineages (CD71) were also reduced in EDAG overexpressing cells.

We next determined whether EDAG confers growth advantage to CD34^+^ cells. EDAG or control lentivirus were transduced into CB CD34^+^ cells and nonsorted cells were cultured in liquid cytokine-driven stroma-free conditions. The transduction efficiency in control and EDAG group was 45% and 42% respectively. During the 7-day period, the content of GFP cells in control group remained steady at ~50% (Fig 1E), which was close to initial transduction efficiency of 45%. Significantly, EDAG-transduced cells (42% transduction efficiency) exhibited a significant growth advantage to total cells, with more than 80% of the cells expressed GFP (Fig 1E).

We next asked if EDAG expression increases clonogenic progenitor activity. We found that total number of colonies formed by EDAG-transduced cells was more than 2-fold higher than that of control cells (Fig 1F). Significantly increased series-plating efficiency was also observed with EDAG-transduced CB CD34^+^ cells compared with control cells. In addition, EDAG-expressing CD34^+^ cells generally formed larger colonies than control cells did (data not shown). We further determined the effect of EDAG on stem cell frequencies by LTC-IC assay in bulk. As shown in Fig 1G, EDAG overexpression led to a strong increase in stem cell frequency.

To exclude the impact of EDAG-GFP fusion on EDAG function, we used pBPLV vector which has two CMV promoters and an IRES-GFP tag. The recombinant vector pBPLV-EDAG expresses EDAG protein and GFP protein simultaneously without fusion. Then CD34^+^ cells were transduced with pBPLV-EDAG or control lentivirus and GFP^+^ cells were sorted. As shown in S2A Fig, EDAG overexpression promoted the cell proliferation dramatically. CFC analysis also suggested that EDAG overexpression increased the clonogenic progenitor activity significantly (S2B Fig).

These results suggest that overexpression of EDAG promotes the proliferation and maintenance of human CB CD34^+^ cells.

### EDAG knockdown decreases the proliferation of human CB CD34^+^ cells

**Fig 2:**
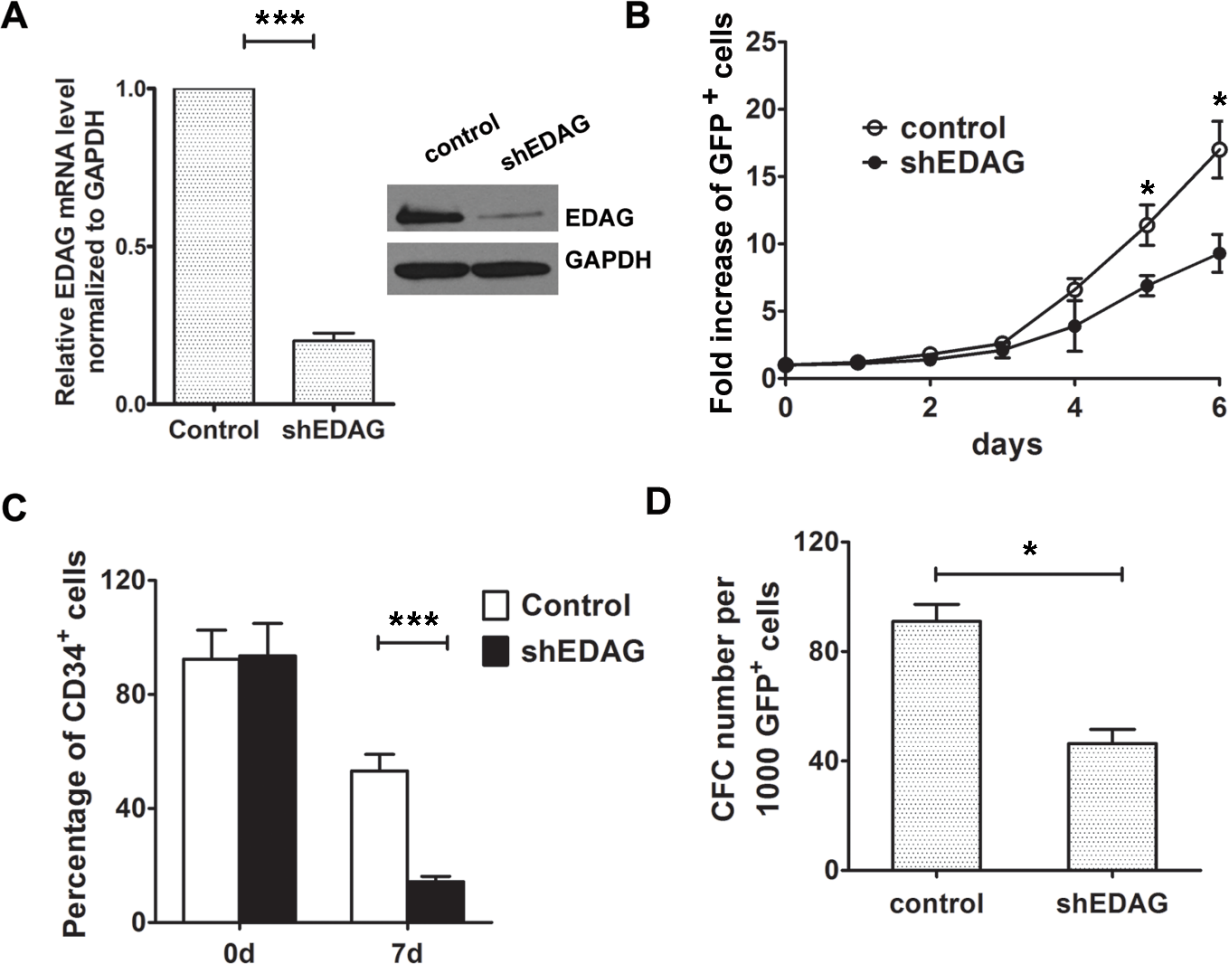
EDAG knockdown decreases the proliferation of human CB CD34^+^ cells. (A) EDAG shRNA or control lentivirus were transduced into CD34^+^ cells and the mRNA expression level of EDAG was investigated using Realtime RT-PCR (left panel). The protein level of EDAG was determined using Western blot (right panel). GAPDH was used as internal control. (B) EDAG shRNA or control lentivirus were transduced into CD34^+^ cells and the sorted GFP^+^ cells were cultured in liquid culture medium with cytokines for the indicated time and the number of cells was counted. The expression of CD34 was analyzed by FACS (C). (D) CFC activity of CB CD34^+^ cells transduced with EDAG shRNA or control lentivirus (Mean ± SD of at least 3 independent experiments). Data are shown as the mean ± S.D and are representative of three independent experiments. * *p* < 0.05, *** *p* < 0.001.

To confirm the effect of EDAG on CD34^+^ cells proliferation, specific shRNA targeted EDAG was used as previously described[10]. EDAG shRNA lentivirus or control lentivirus were transduced into CD34^+^ cells and then the sorted GFP^+^ cells were cultured in liquid culture medium with cytokines (Fig 2A). As shown in Fig 2B, EDAG knockdown led to a significant reduction of cell number within 6 days culture. The percentage of CD34^+^ cells was also decreased compared to the control group (Fig 2C). Furthermore, CFC analysis of EDAG knockdown cells suggested that EDAG down-regulation resulted in a significant decrease in the total number of CFC cells after 2 weeks (Fig 2D). The similar result was obtained in secondary replating experiment which is consistent with our previous study[10].

Taken together, these results indicate that EDAG promotes the expansion of human CB CD34^+^ cells in liquid cultures and is essential for maintenance of CD34^+^ cells.

### EDAG overexpression increases HSC repopulating capacity

**Fig 3:**
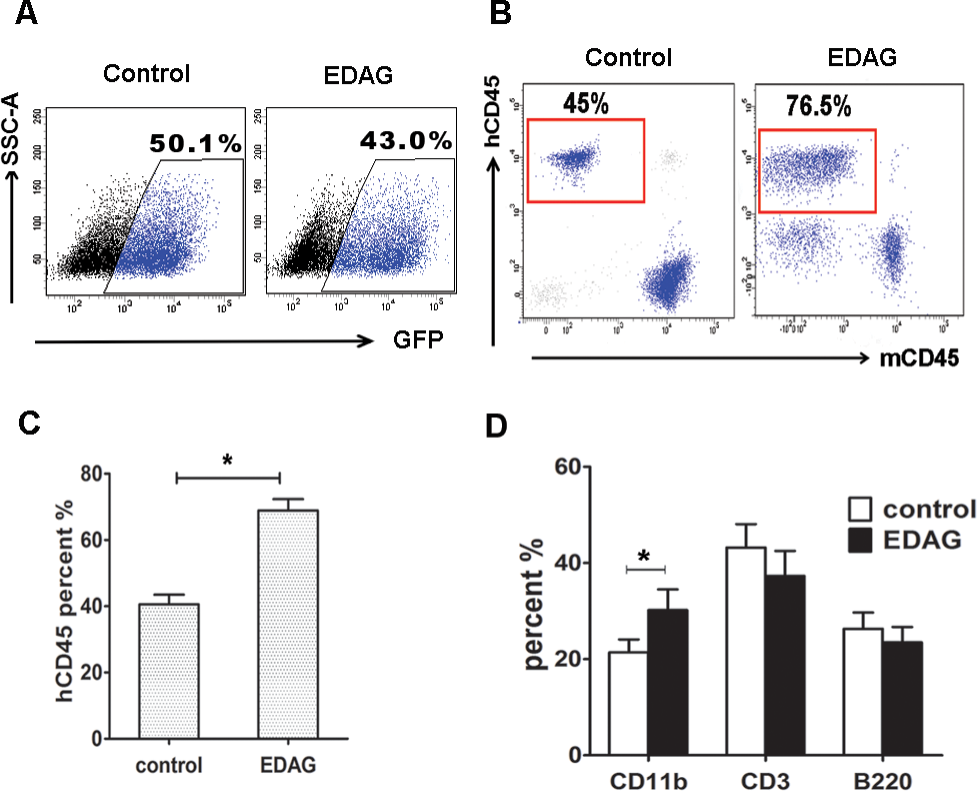
EDAG overexpression increases HSC repopulating capacity. (A) Human CD34^+^ cells were cultured for 24 hours in medium containing cytokines and then infected with EDAG or control lentivirus. The GFP percentage was measured by FACS anlaysis. Then sorted GFP^+^ cells were injected intravenously into sublethally irradiated NOD/SCIDIL2Rγ^null^ mice. Engraftment was determined at 16 weeks by analysis of human CD45^+^ cells (hCD45) in peripheral blood, the mouse CD45 was used as internal control (B-C). The percentage of different lineages was determined by analysis of multilineage markers (B). The data were shown as the mean ± SD and are representative of three independent experiments. * *p* < 0.05.

We further performed CB CD34^+^ cells transplantation to evaluate the *in vivo* function of EDAG-expressing hematopoietic progenitor cells. CD34^+^ cells were transduced with EDAG or control lentivirus and then injected directly into sublethally irradiated NOD/SCIDIL2Rγ^null^ recipients. The initial transduction efficiency of GFP in EDAG-transduced cells is comparable to that in control cells (43% vs 50%, Fig 3A). At 16 weeks after transplantation, the mice were sacrificed and the chimerism was analyzed. Significantly, EDAG-transduced cells constituted more than 76.5% of the peripheral blood cells, compared with less than 45% reconstitution by control cells (Fig 3B and 3C). The similar result was detected in bone marrow. Examination of lineage distribution of GFP positive cells in the peripheral blood cells from the 16-week post-transplanted mice shows that CD34^+^ cells overexpressing EDAG has the ability of multilineage reconstitution with increased myeloid (CD11b^+^) cells (Fig 3D). Hence, EDAG overexpression increases the long-term repopulating capacity of HSPCs.

### EDAG enhances survival and prevents apoptosis of human CB CD34^+^ cells

**Fig 4:**
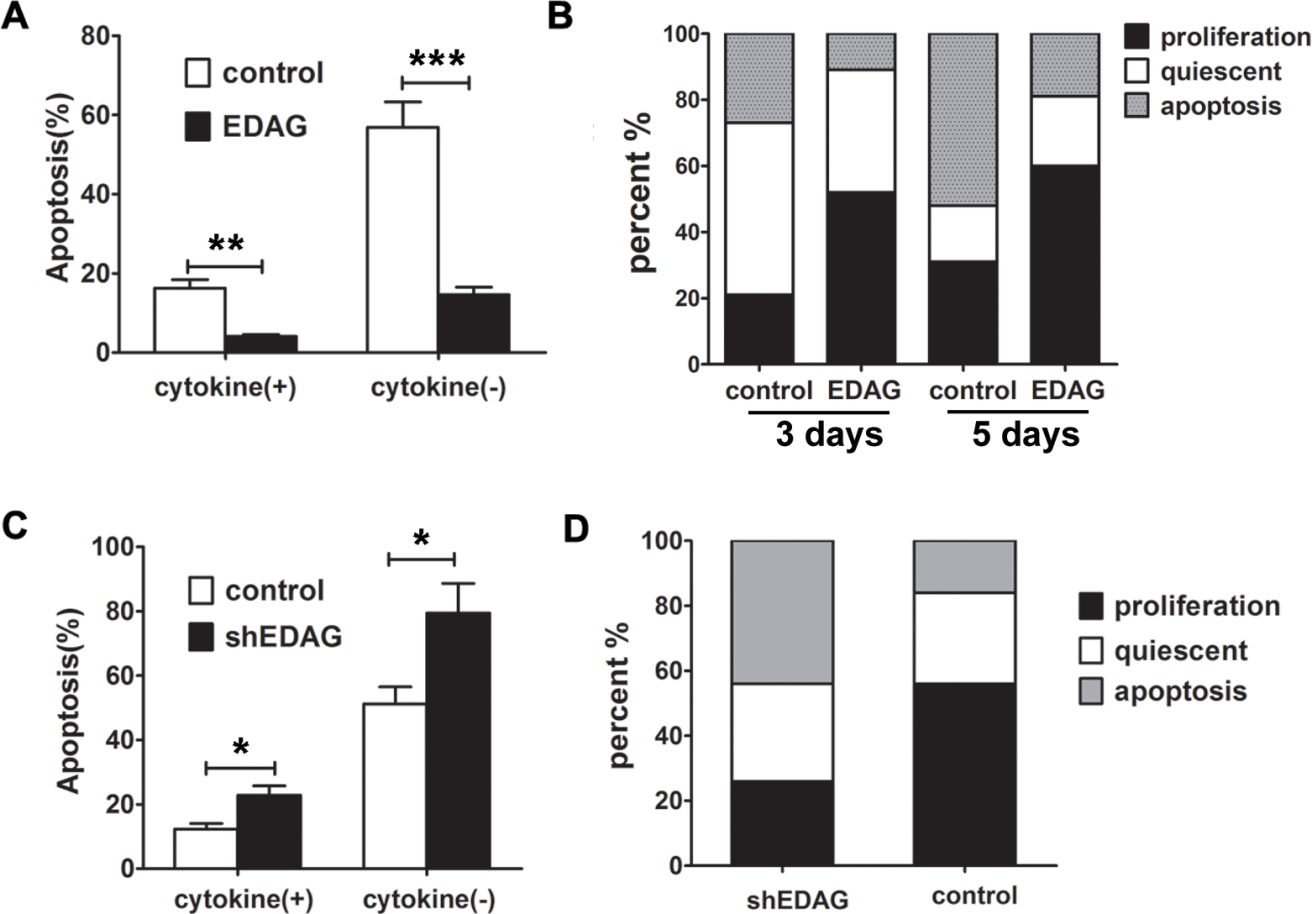
EDAG enhances survival and prevents apoptosis of human CB CD34^+^ cells. (A) CB CD34^+^ cells were transduced with EDAG or control lentivirus and GFP^+^ cells were sorted and cultured in liquid cytokine-driven conditions for 10 days. The cytokines were deprived from the cultured cells for 24 hours and cell apoptosis was detected using AnnexinV-APC and PI staining by FACS. (B) CB CD34^+^ cells were transduced with control or EDAG lentivirus and GFP^+^ cells were sorted. Single cells were deposited in 96-well plates and cultured in stroma-free conditions in medium supplemented with a cocktail of cytokines. Wells were evaluated microscopically every day. (C) EDAG shRNA or control lentivirus were transduced into CD34^+^ cells and cultured for 10 days. The cytokines were deprived from the cultured cells for 24 hours and cell apoptosis was detected. (D) Single-cell analysis of EDAG shRNA or control lentivirus transduced CD34^+^ cells. The data were shown as the mean ± SD and are representative of three independent experiments. * *p*<0.05, ** *p* < 0.01, *** *p* < 0.001.

Since EDAG expression promotes the proliferation and maintains stem-cell characteristics of CB CD34^+^ cells, we examined the effect of EDAG on cell survival and apoptosis. CB CD34^+^ cells were transduced with EDAG or control lentivirus and GFP^+^ cells were sorted and cultured in liquid cytokine-driven conditions for 10 days. In control group, ~16% of cells were apoptotic and in EDAG overexpressing cells, no obvious apoptosis was detected (Fig 4A). When the cytokines were deprived for 24 hour, the cell apoptosis in control group was significantly increased to about 56% and EDAG overexpression reduced the cell apoptosis significantly.

We further detected the cell survival at single-cell level. CB CD34^+^ cells were transduced with EDAG or control virus and GFP^+^ cells were sorted. Single cells were deposited in 96-well plates and cultured in stroma-free conditions in medium supplemented with a cocktail of cytokines. The wells were scored for the presence of living cells at different time points. When a single cell was seen in the well, it was referred to as “quiescent”; when 2 or more cells were observed, the well was referred to as “proliferation”; and if no cells were seen, it was referred to as “apoptosis”. After 3 days, in control group ~21% of the wells were proliferating cells and ~27% cells died, and in EDAG group, the proliferating cells were 52% and the apoptosis rate was 11% (Fig 4B). At day 5, in control group, the proliferating cells rate was 31% and the apoptosis was increased to 52%, while in EDAG group, about 60% wells were proliferating cells and only 19% of the wells contained apoptotic cells.

To confirm the effect of EDAG on cell survival, EDAG shRNA or control lentivirus were transduced into CD34^+^ cells and cultured for 10 days. A strongly increased levels of apoptosis was observed as determined by propidium iodide (PI)/annexinV staining (Fig 4C). When the cytokines were deprived for 24 hour, the cell apoptosis in EDAG knockdown cells significantly increased compared to control cells. Single-cell analysis was further performed. As shown in Fig 4D, after 4 days culture, the number of apoptotic wells remained significantly higher in the cells with EDAG knockdown.

These data suggest that EDAG prevents apoptosis and promotes the proliferation and survival of individual HSPC cells.

### EDAG is a positive regulator of HSC cell cycle progression

**Fig 5:**
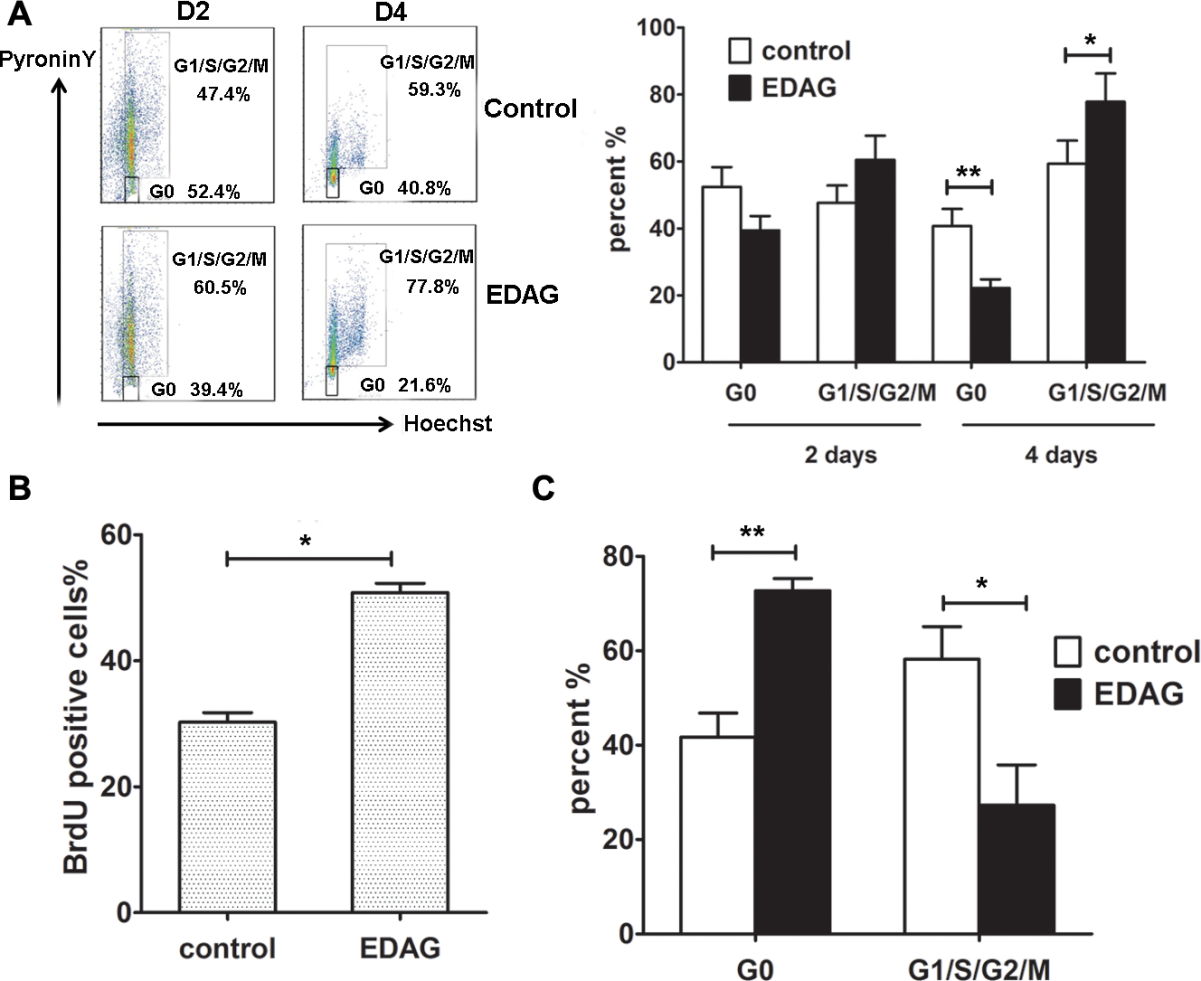
EDAG is a positive regulator of HSC cell cycle progression. (A) CB CD34^+^ cells were infected with control or EDAG lentivirus. GFP^+^ cells were sorted and cultured in liquid medium containing cytokines for the indicated time. Then Cells were harvested and stained by Hoechst 33342 and PyroninY for cell cycle analysis. BrdU labeling was performed to determine the percentage of S-phase cells (B). (C) EDAG shRNA or control lentivirus were transduced into CD34^+^ cells and cultured in liquid stroma-free conditions for 4 days. Then the cell cycle distribution was performed by FACS. The data were shown as the mean ± SD and are representative of three independent experiments. * *p* < 0.05, ** *p* < 0.01.

To address how EDAG enhances HSC and progenitor cell expansion, we analyzed cell cycle profile of EDAG-transduced CB CD34^+^ cells. Co-staining of Hoechst 33342 and Pyronin Y was used to identify G0 cells from G1 cells in hematopoietic stem cells. As shown in Fig 5A, after 2 days culture, EDAG overexpression prominently reduced cells in the G0 phase (39.4% compared with 52.4% in control cells). This coincided with a significant increase in the cell cycle entry (G1/S/M, 60.5% compared with 47.4% in control cells). After 4 days culture, in EDAG group, more cells were induced to enter cell cycle with significantly increased G1/S/G2/M phase percentage (77.8% compared with 59.3% in control cells). BrdU labeling experiments show that the percentage of cells entering the cell cycle was significantly higher in EDAG-transduced cells than in vector-transduced cells (49% compared with 32%) (Fig 5B).

To confirm the effect of EDAG on cell cycle of CD34^+^ cells, EDAG shRNA or control lentivirus were transduced into CD34^+^ cells and cultured in liquid stroma-free conditions. Consistently, after 4 days culture, EDAG knockdown led to a significicant reduction of cells in G1/S/G2/M phase compared with control (27.3% vs 58.2%) (Fig 5C).

This data suggest that EDAG induces a rapid cell cycle entry of CB CD34^+^ cells.

### EDAG regulates the expression of several positive cell cycle regulators

**Fig 6:**
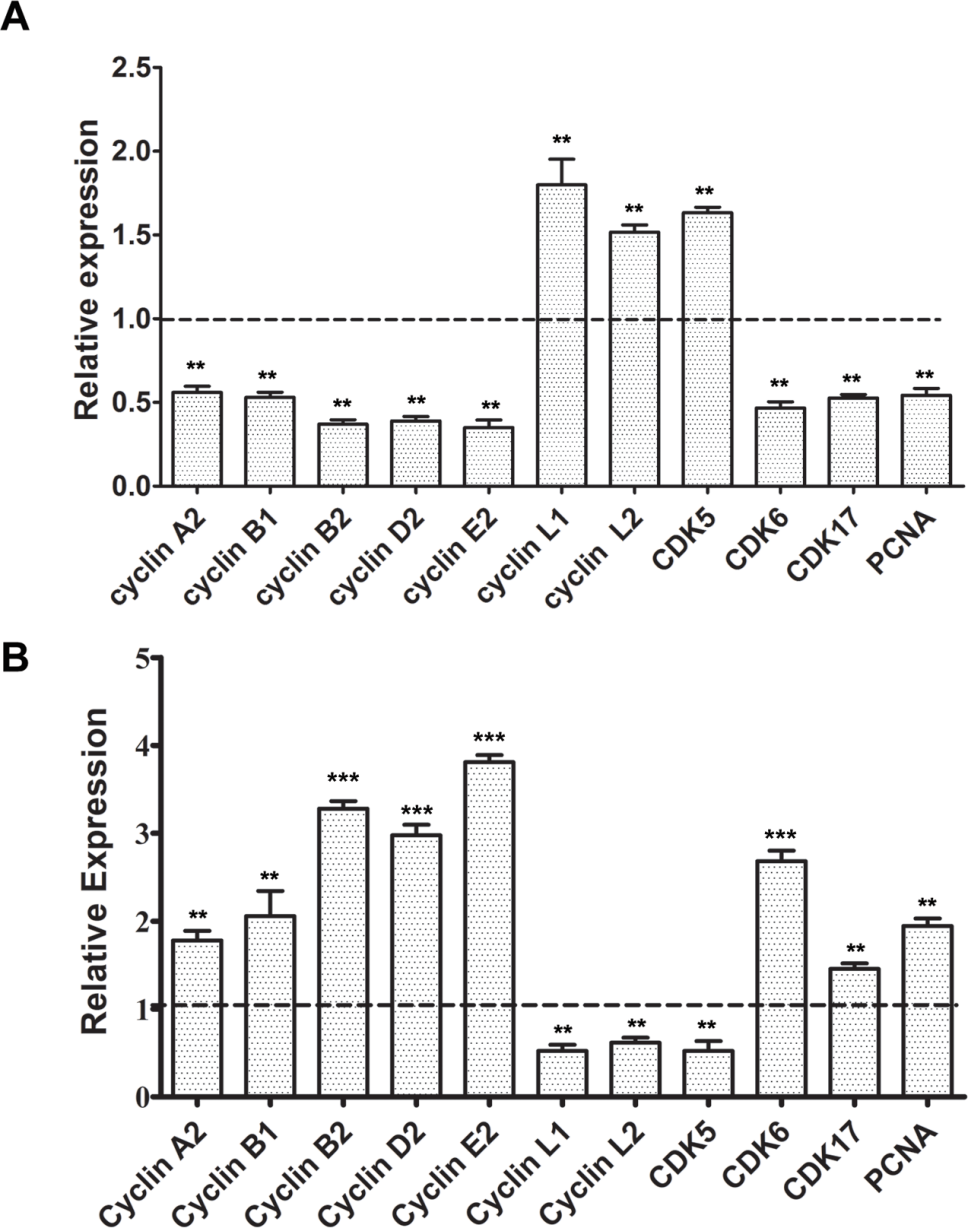
EDAG regulates the expression of several positive cell cycle regulators. (A) CD34^+^ cells were infected with EDAG shRNA or control lentivirus and cultured for 2 days. (B) CD34^+^ cells were infected with EDAG overexpressing lentivirus or control lentivirus and cultured for 2 days. Then total RNA was extracted for Realtime RT-PCR analysis. The data were normalized to GAPDH expression (Mean ± SD of 3 independent experiments). ** *p* < 0.01.

In our previous study, using microarray assay we find that EDAG down-regulation led to alterations of various cell cycle related genes, including cyclins (cyclin A2, B1, B2, D2, E2, L1, L2), cyclin-dependent kinases (CDK5, CDK6, CDK17), and PCNA. To investigate the molecular mechanism whereby EDAG promotes HSC expansion and cell cycle progression, we examined the gene expression profiling by Realtime RT-PCR in control and EDAG shRNA lentivirus-transduced CD34^+^ cells. As shown in Fig 6A, the expression of cyclin A2, B1, B2, D2, E2, PCNA and CDK6 were significantly down-regulated with EDAG knockdown. To confirm the effect of EDAG on these genes, we performed Realtime RT-PCR analysis in EDAG or control lentivirus-transduced CD34^+^ cells. Consistently, the expression of cyclin A2, B1, B2, D2, E2, PCNA and CDK6 were significantly increased with EDAG overexpression (Fig 6B). These data suggested that EDAG regulates the cell cycle probably via modulating the expression the cell cycle regulators.

## Discussion

EDAG has been considered to be an important gene that controls different aspects of hematopoietic cells, ranging from hematopoietic cell differentiation to the control of apoptosis[9, 12–15]. Although previous study suggests that EDAG is highly expressed in HSPC, the role of EDAG in HSPC expansion and survival remains unknown. Our present study demonstrate that EDAG promotes the expansion of human CD34^+^ cells *ex vivo*, increases survival, prevents apoptosis and enhances their repopulating capacity. Mechanistic analysis suggests that EDAG induces rapid entry of CD34^+^ cells into the cell cycle and regulates the expression of cell cycle regulators. In this way, our results suggest that EDAG is essential for the expansion and survival of human HPSC.

Due to the limited CD34^+^ cell number in one single cord blood unit, much of the current efforts are focusing on developing technology of *ex vivo* expansion of HSPC. Many extrinsic factors have been identified as regulators of HSPC, such as cytokines[16], Notch ligands[17], Wnt ligands[18] and Angiopoietins[19]. However, only a few of transcription factors/regulators have been reported to positively regulate ex vivo HSC expansion. The transcription factor HOXB4 overexpression lead to expansion of murine as well as human HSPC[20, 21]. Expressing a HOXB4 fusion protein that is passively taken up by the CD34^+^ cells in a stromal cell layer result in a 2.5-fold increase in long-term repopulating cells compared with control[22]. The POU domain family transcriptional factor Oct4 is a master regulator for maintenance of totipotency and pluoripotency[23]. Activation of Oct4 by Oct4-activating compound 1 (OAC1) in CB CD 34^+^ cells enhanced ex vivo expansion of HSPC by regulating HOXB4 expression[24]. Constitutive expression of the Polycomb group transcriptional repressor, BMI1, in human CB CD34^+^ cells results in prolonged maintenance of the stem-cell pool and enhances self-renewal of human stem and progenitor cells[25]. Repression of BMI1 led to impaired long-term expansion and progenitor-forming capacity with enhanced apoptosis, indicating that BMI1 expression is required for maintenance and self-renewal of HSPC[26]. Here, we demonstrate that the EDAG is another transcriptional regulator that has an important role of HSPS expansion. Using both ex vivo and transplantation models, we observed a significant increase in the number of HSPC and long-term HSC in vivo repopulating capability.

Previous study suggest that rapid exit from G0/G1 phases of cell cycle in response to stem cell factor confers on umbilical cord blood CD34^+^ cells an enhanced ex vivo expansion potential[27]. Our study shows that EDAG overexpression induces a rapid entry of CD34^+^ cells into cell cycle, from G0 phase to G1/S/M phase. The positive cell cycle regulators such as cyclin A2, B1, B2, D2, E2, PCNA and CDK6 are all significantly down-regulated by EDAG knockdown. Previous study report that cyclin A in cultured cells affects cell cycle progression and leads to accelerated entry into S phase[28]. Cyclin A2 is postulated to play a role in entry of cells into mitosis[29] and essential for the proliferation of HSC[30]. Inhibition of cyclin A2 function by p21^Cip1^ during the G_2_ phase blocks G_2_-M phase progression[31, 32]. Cyclin B1 plays a central role in the G_2_/M transition of the cell cycle and has also been implicated in apoptosis[33]. Cyclin D2 is highly expressed in all hematopoietic progenitors[34] and decreased expression of cyclin D2 is necessary for terminal myeloid differentiation[35, 36]. Cyclin E2 is the partner of CDK2[37] and promotes progression of the cell cycle from G_1_ to S phase[38]. CDK6 has been reported to regulate quiescence exit in human HSC and enforced CDK6 expression in LT-HSCs shortens quiescence exit and confers competitive advantage without impacting function[39]. More interestingly, ChIP-Seq analysis suggest that CD34^+^ cells EDAG occupies 7133 cis-regulatory elements corresponding to 3847 genes, which includes CDK6. So it is possible that EDAG regulates CDK6 expression through direct binding to its promoter region. All these data suggest that the enhanced proliferative effect of EDAG might due to its ability to induce rapid entry of HPSC into the cell cycle, probably via regulating the expression of positive cell cycle regulators. However, how EDAG regulates these genes expression needs further investigation.

In summary, here we demonstrate that EDAG is a novel important intrinsic factor for *ex vivo* HSPC expansion and maintenance.

## Supporting information

S1 Fig Densitometry analysis of the endogenous EDAG immunoblot bands in Fig 1A. (DOCX)

S2 Fig EDAG overexpression promotes the proliferation and aintenance of human CB CD34^+^ cells. pBPLV-EDAG or control lentivirus were transduced into CB CD34^+^ cells and GFP-positive cells were sorted. Then cells were plated in liquid cultures for the indicated time and the number of cells was counted. (B) CFC activity of CB CD34^+^ cells transduced with EDAG or control lentivirus with serial replating. Data are shown as the mean ± S.D and are representative of three independent experiments. ** *p* < 0.01.
(DOCX)

S1 Table Sequences of primers used in the present study. (DOCX)
