# Supplementary figures and images for: EDAG promotes the expansion and survival of human CD34+ cells
Source: PLoS One. 2018 Jan 11;13(1):e0190794. doi: 10.1371/journal.pone.0190794 (PMC5764277; doi:10.1371/journal.pone.0190794)

**
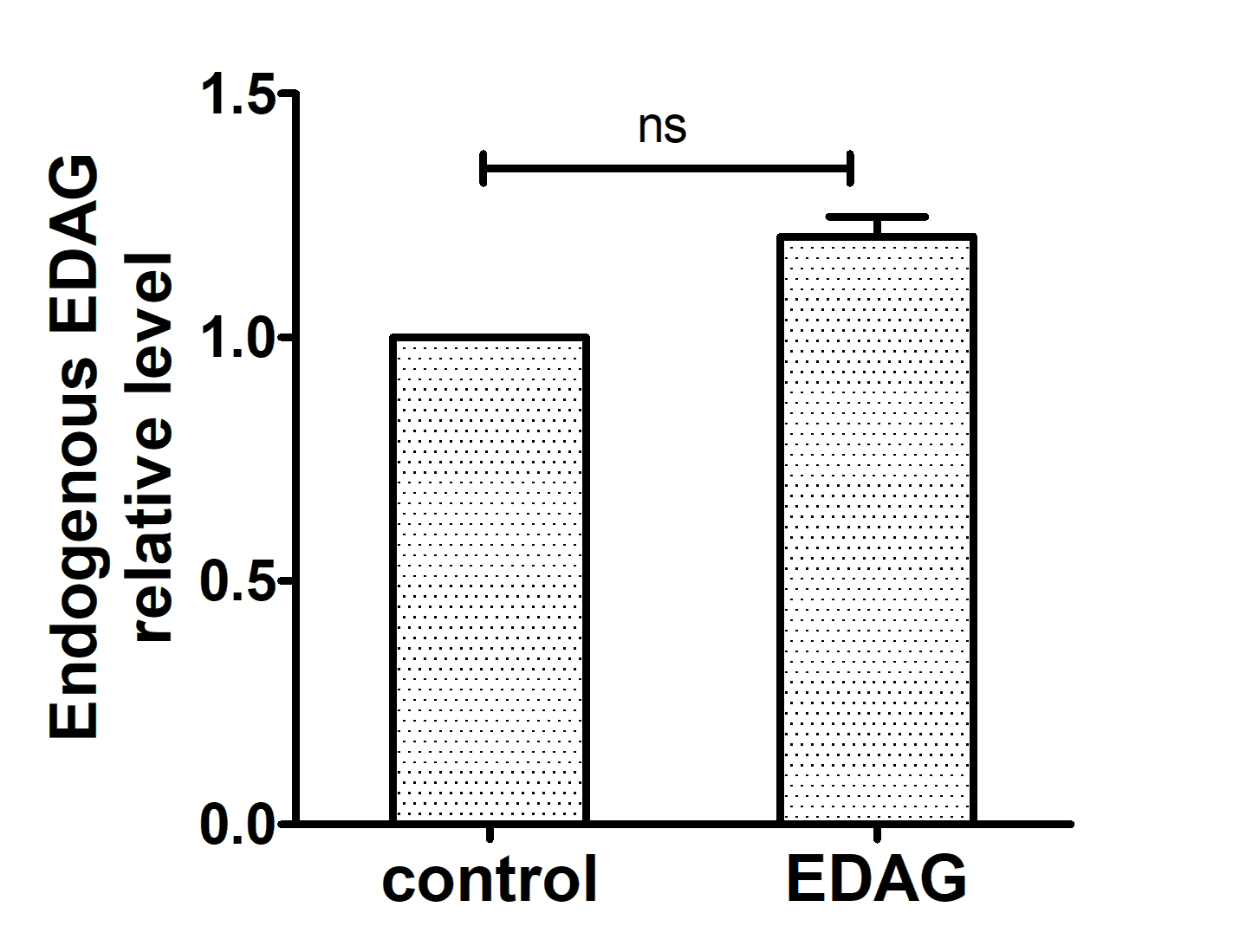
**

**Fig S1. Densitometry analysis of the endogenous EDAG immunoblot bands in Fig 1A.**

Supplement: S1 Fig — (DOCX) [file pone.0190794.s001.docx]
