# Supplementary material for: EDAG promotes the expansion and survival of human CD34+ cells
Source: PLoS One. 2018 Jan 11;13(1):e0190794. doi: 10.1371/journal.pone.0190794 (PMC5764277; doi:10.1371/journal.pone.0190794)
Supplement: S2 Fig — pBPLV-EDAG or control lentivirus were transduced into CB CD34+ cells and GFP-positive cells were sorted. Then cells were plated in liquid cultures for the indicated time and the number of cells was counted. (B) CFC activity of CB CD34+ cells transduced with EDAG or control lentivirus with serial replating. Data are shown as the mean ± S.D and are representative of three independent experiments. ** p < 0.01. (DOCX) [file pone.0190794.s002.docx]

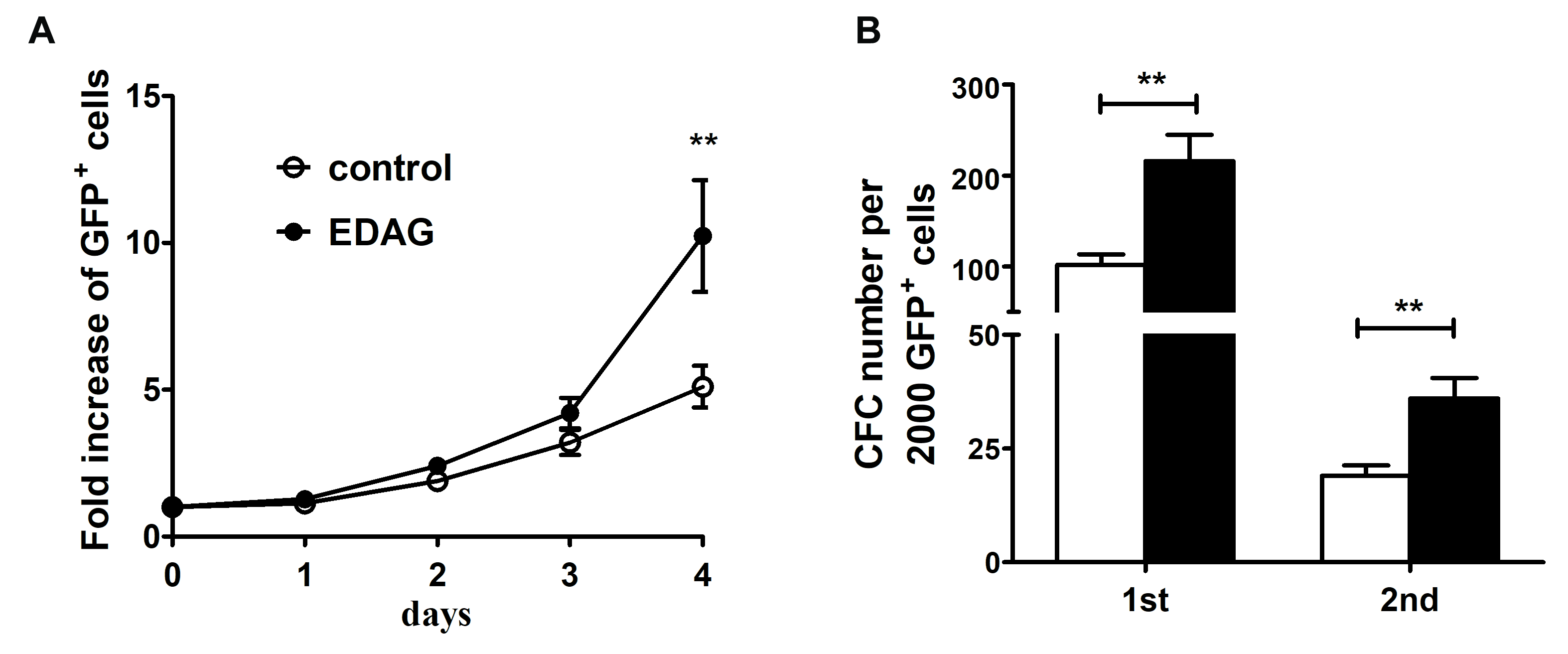


**Fig S2. EDAG overexpression promotes the proliferation and aintenance of human CB CD34^+^ cells.** pBPLV**-**EDAG or control lentivirus were transduced into CB CD34^+^ cells and GFP-positive cells were sorted. Then cells were plated in liquid cultures for the indicated time and the number of cells was counted. (B) CFC activity of CB CD34^+^ cells transduced with EDAG or control lentivirus with serial replating. Data are shown as the mean ± S.D and are representative of three independent experiments. ** *p* < 0.01.
