# Supplementary material for: EDAG promotes the expansion and survival of human CD34+ cells
Source: PLoS One. 2018 Jan 11;13(1):e0190794. doi: 10.1371/journal.pone.0190794 (PMC5764277; doi:10.1371/journal.pone.0190794)
Supplement: S1 Table — (DOCX) [file pone.0190794.s003.docx]

**Table S1. Sequences of primers used in the present study**

| Target gene |  | Sequences (5’-3’) | Use |
| --- | --- | --- | --- |
| GAPDH | Forward | TGTTGCCATCAATGACCCCTT | Realtime PCR |
|  | Reverse | CTCCACGACGTACTCAGCG | Realtime PCR |
| EDAG | Forward | TCAGACACCTGACCCTCATCAA | Realtime PCR |
|  | Reverse | GCCAGTGCTTTCTCCACTATTTC | Realtime PCR |
| CDK6 | Forward | CCAGATGGCTCTAACCTCAGT | Realtime PCR |
|  | Reverse | AACTTCCACGAAAAAGAGGCTT | Realtime PCR |
| Cyclin A2 | Forward | GGATGGTAGTTTTGAGTCACCAC | Realtime PCR |
|  | Reverse | CACGAGGATAGCTCTCATACTGT | Realtime PCR |
| Cyclin B1 | Forward | TTGGGGACATTGGTAACAAAGTC | Realtime PCR |
|  | Reverse | ATAGGCTCAGGCGAAAGTTTTT | Realtime PCR |
| Cyclin B2 | Forward | TGCTCTGCAAAATCGAGGACA | Realtime PCR |
|  | Reverse | GCCAATCCACTAGGATGGCA | Realtime PCR |
| Cyclin D2 | Forward | ACCTTCCGCAGTGCTCCTA | Realtime PCR |
|  | Reverse | CCCAGCCAAGAAACGGTCC | Realtime PCR |
| Cyclin E2 | Forward | GGAACCACAGATGAGGTCCAT | Realtime PCR |
|  | Reverse | CCATCAGTGACGTAAGCAAACT | Realtime PCR |
| Cyclin L1 | Forward | AGACGGACTTACGCATCCTG | Realtime PCR |
|  | Reverse | TCTCGAAACTGTGTTTGACGAAA | Realtime PCR |
| Cyclin L2 | Forward | GTACTCCGGGGTGCTCATC | Realtime PCR |
|  | Reverse | GAGGTCGGTCTCTGTGTCG | Realtime PCR |
| PCNA | Forward | ACACTAAGGGCCGAAGATAACG | Realtime PCR |
|  | Reverse | ACAGCATCTCCAATATGGCTGA | Realtime PCR |
| CDK5 | Forward | TTTTTCCCGGCAATGATGTCG | Realtime PCR |
|  | Reverse | GCAGCTTGGTCATAGAGGGC | Realtime PCR |
| CDK17 | Forward | CATAGACGGATCTCAATGGAGGA | Realtime PCR |
|  | Reverse | TGGTTGGTCAAATGGTGGACT | Realtime PCR |
